# Retraction: Novel insights into the role of mitochondria-derived peptides in myocardial infarction

**DOI:** 10.3389/fphys.2022.1006441

**Published:** 2022-08-04

**Authors:** Frontiers Editorial Office

The Publisher retracts the cited article.

Following publication, the publisher uncovered conclusive evidence that false identities were used for one of the authors and for both of the reviewers of this article. The investigation, conducted in accordance with Frontiers’ policies and the Committee on Publication Ethics (COPE) guidelines, revealed that the article was submitted using the false identity Enny Kampmann. We were able to confirm with the administrators that no one of this name has ever been involved with the City College of San Francisco, the purported affiliation.

Queries to the co-authors and editors were not able to resolve the matter and the publisher retracts the article.

This retraction was approved by the Chief Editors of Frontiers in Physiology and the Chief Executive Editor of Frontiers.

